# Hydroxypropyl Cellulose Based Non-Volatile Gel Polymer Electrolytes for Dye-Sensitized Solar Cell Applications using 1-methyl-3-propylimidazolium iodide ionic liquid

**DOI:** 10.1038/srep18056

**Published:** 2015-12-11

**Authors:** Mohammad Hassan Khanmirzaei, S. Ramesh, K. Ramesh

**Affiliations:** 1 Centre for Ionics University of Malaya, Department of Physics, Faculty of Science, University of Malaya, 50603 Kuala Lumpur, Malaysia

## Abstract

Gel polymer electrolytes using imidazolium based ionic liquids have attracted much attention in dye-sensitized solar cell applications. Hydroxypropyl cellulose (HPC), sodium iodide (NaI), 1-methyl-3-propylimidazolium iodide (MPII) as ionic liquid (IL), ethylene carbonate (EC) and propylene carbonate (PC) are used for preparation of non-volatile gel polymer electrolyte (GPE) system (HPC:EC:PC:NaI:MPII) for dye-sensitized solar cell (DSSC) applications. The highest ionic conductivity of 7.37 × 10^−3^ S cm^−1^ is achieved after introducing 100% of MPII with respect to the weight of HPC. Temperature-dependent ionic conductivity of gel polymer electrolytes is studied in this work. XRD patterns of gel polymer electrolytes are studied to confirm complexation between HPC polymer, NaI and MPII. Thermal behavior of the GPEs is studied using simultaneous thermal analyzer (STA) and differential scanning calorimetry (DSC). DSSCs are fabricated using gel polymer electrolytes and J-V centeracteristics of fabricated dye sensitized solar cells were analyzed. The gel polymer electrolyte with 100 wt.% of MPII ionic liquid shows the best performance and energy conversion efficiency of 5.79%, with short-circuit current density, open-circuit voltage and fill factor of 13.73 mA cm^−2^, 610 mV and 69.1%, respectively.

The investigations on dye-sensitized solar cells have dramatically increased in recent decades due to low cost, easy fabrication and carbon free advantage of (DSSCs)^1^,^2^. One of the big challenges for DSSC fabrication is electrolyte preparation. Among the electrolytes, liquid electrolyte is widely used for DSSC fabrication but they have some disadvantages such as liquid linkage and corrosion. One method to overcome this issue is through usage of gel electrolytes^3^,^4^. Gel polymer electrolytes (GPEs) have also been extensively investigated for DSSC fabrication^5^,^6^,^7^,^8^,^9^. Therefore, gel polymer electrolytes (GPEs) are a good alternative for DSSC applications because of advantages such as low vapor pressure, excellent contacting and filling properties between the nanostructured electrode and counter electrode, higher ionic conductivity compared to the conventional polymer electrolytes, excellent thermal stability and outstanding long-term stability^10^. Consequently, cellulose based polymer electrolytes are incorporated in several researches for electrochemical applications^11^,^12^,^13^,^14^ including DSSC applications due to good mechanical performance and thermal stability^15^.

Ionic Liquids (ILs) are good candidates as plasticizers and liquid salts for gel polymer electrolytes and electrochemical applications due to their negligible vapor pressure, non-inflammability, excellent chemical and thermal stability and high ionic conductivity^16^. Among the ionic liquids, imidazolium iodide based ionic liquids are widely used for dye-sensitized solar cell (DSSC) applications because of better performance^17^,^18^,^19^,^20^,^21^. Some kinds of imidazolium based ionic liquids or electrolytes synthesized by UV-cured^22^ procedure or in some novel ionogel forms^23^ can be used in DSSCs as well. Furthermore, 1-methyl-3-propylimidazolium iodide (MPII) provides excellent efficiency and good stability in dye-sensitized solar cells^24^.

In this work, hydroxylpropyl cellulose, sodium iodide and MPII were used to prepare gel polymer electrolytes. Ionic conductivity and temperature-dependent conductivity studies were carried out using electrochemical impedance spectroscopy (EIS). Structural centeracterization was performed using X-Ray diffraction (XRD). Thermal behaviors of samples were studied using simultaneous thermal analyzer (STA) and differential scanning calorimetry (DSC). The gel polymer electrolyte based dye-sensitized solar cells were fabricated and tested under Sun simulator.

## Results and Discussion

**Figure 1:**
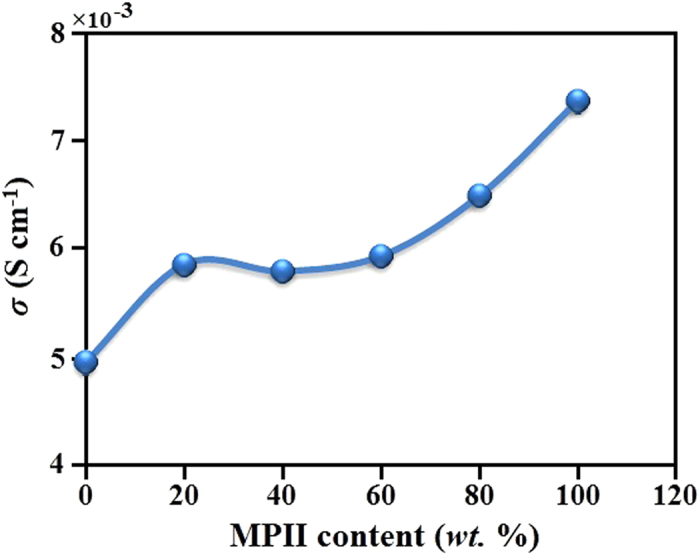
Variation of Ionic conductivity with MPII ionic liquid content.

**Figure 2:**
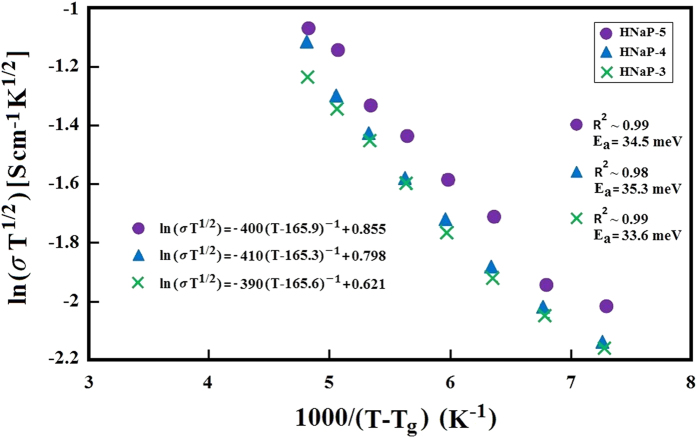
Temperature-dependence ionic conductivity of HPC:EC:PC:NaI:MPII GPE system.

The variation of the ionic conductivity with MPII ionic liquid content is exhibited in Fig. 1. The highest ionic conductivity of 7.37 × 10^−3^ S/cm was achieved after addition of 100 wt.% of MPII ionic liquid (HNaP-5). Table 1 shows the ionic conductivity values for all GPEs. The results show that ionic conductivity dramatically increased after addition of MPII ionic liquid. This can be due to the increase of mobile ions after incorporation and addition of MPII ionic liquid. On the other hand, due to the high self-dissociating and ion-transporting abilities of the constituent ionic liquid, its conductivity increases with the carrier numbers in the polymer electrolytes. Besides that, the increase of the amount of the ionic liquid weakens the interaction among the polymer chains, accelerates the decoupling of the ion transport from polymer segmental motion^25^. Figure 2 demonstrates temperature-dependent results for the gel polymer electrolytes. The system follows Vogel–Tammann–Fulcher (VTF) model and were fitted to the VTF equation^26^,^27^


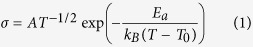


where 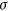 is ionic conductivity, *T* is the absolute temperature, 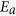 is activation energy, A is pre-exponential factor, *T*_*0*_ is the reference temperature related to the equilibrium state glass transition temperature and *k*_*B*_ is the Boltzmann constant. The regressions (R2~0.99) denotes that the results are almost fitted to the VTF equation. The activation energies have been calculated and inserted in Fig. 2.

**Figure 3:**
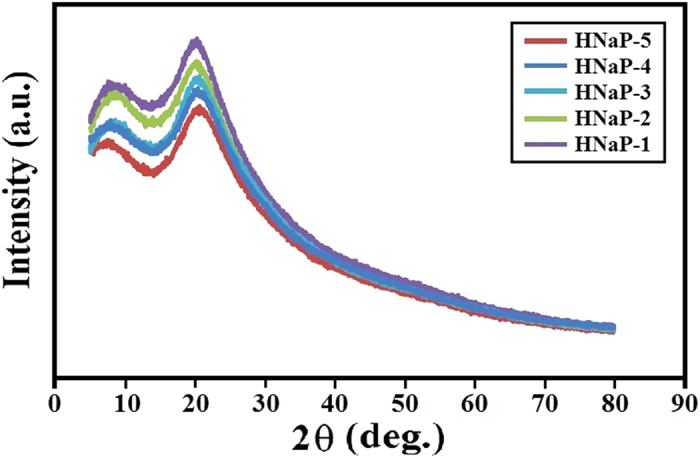
XRD patterns of GPEs for HNaP-1, HNaP-2, HNaP-3, HNaP-4 and HNaP-5.

The XRD patterns of HPC:EC:PC:NaI:MPII system are represented in Fig. 3. The graph shows XRD patterns of HNaP-1, HNaP-2, HNaP-3, HNaP-4 and HNaP-5. The XRD patterns of GPEs show decrease of intensity with the addition of MPII ionic liquid content. The lowest intensity is achieved with incorporation of 100 wt.% of MPII ionic liquid. Consequently, the variation of intensity in gel polymer electrolytes after addition of MPII can be expected as the evidence of complexation between HPC polymer and MPII ionic liquid.

**Figure 4:**
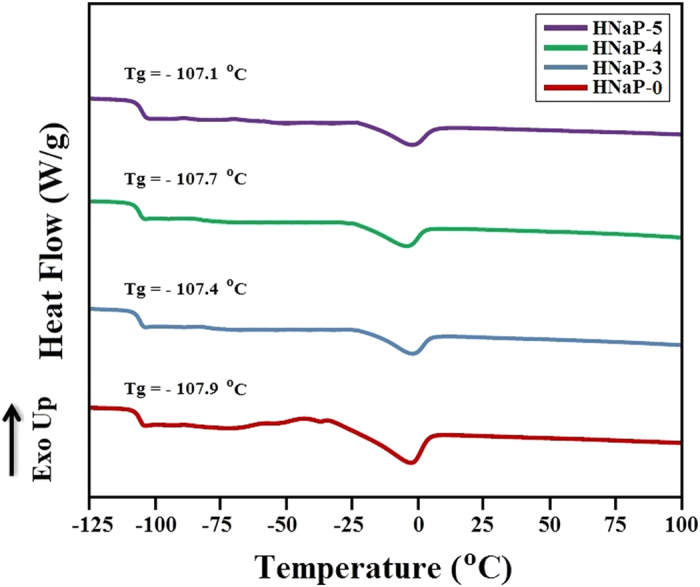
DSC thermograms for HNaP-0, HNaP-3, HNaP-4 and HNaP-5.

Thermal behavior of HPC:EC:PC:NaI:MPII GPE system was studied using simultaneous thermal analyzer (STA) and differential scanning calorimetry (DSC). The HNaP-3, HNaP-4 and HNaP-5 GPEs were analyzed using STA and thermograms show decomposition temperature (T_dc_) of 151, 154 and 155 °C, respectively. The STA thermograms show a slight increase in T_dc_ upon addition of MPII ionic liquid. Figure 4 illustrates the thermal analysis results of DSC for HNaP-0, HNaP-3, HNaP-4 and HNaP-5. The glass transition temperature (Tg) for HNaP-0, HNaP-3, HNaP-4 and HNaP-5 performed using DSC were −107.9, −107.4, −107.7 and −107.1 °C, respectively. The DSC thermographs show that the T_g_ is almost unchanged after addition of MPII ionic liquid which is around −107 °C. Since the increase in ionic conductivity with addition of MPII (according to Fig. 1) may be due to increasing number of centerge carriers, therefore the T_g_ became almost unchanged in different MPII weight ratios^27^. The thermal results show the effect of MPII ionic liquid on GPE system with complexation between HPC, NaI and MPII.

**Figure 5:**
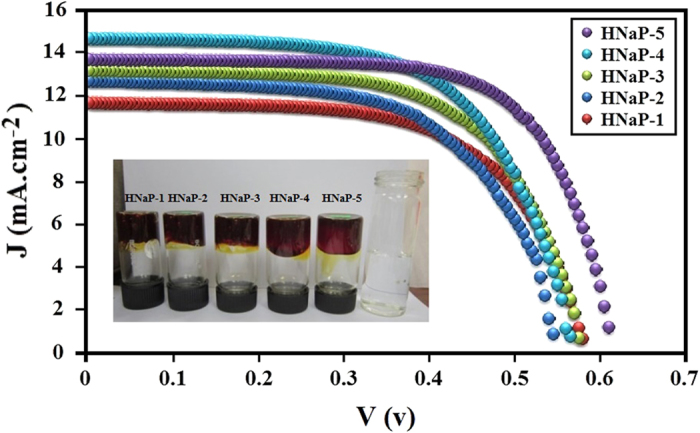
Photocurrent density versus cell potential (J–V) for DSSC cells using HNaP-1, HNaP-2, HNaP-3, HNaP-4 and HNaP-5.

**Figure 6:**
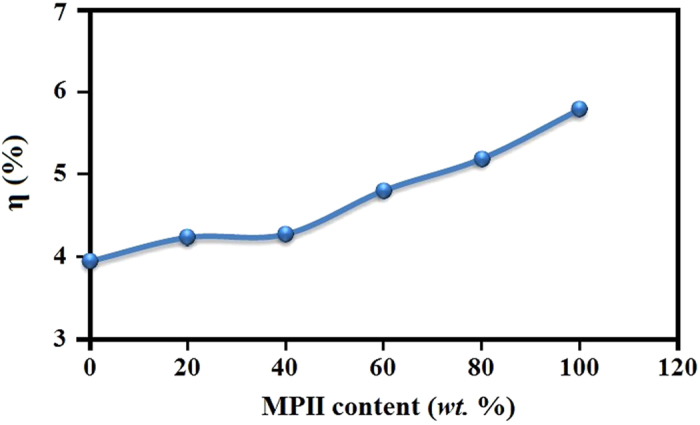
Variation of energy conversion efficiency with MPII ionic liquid content.

The GPEs were sandwiched between two anode and cathode to fabricate DSSC cell. The DSSC cells were fabricated with HNaP-1, HNaP-2, HNaP-3, HNaP-4 and HNaP-5. The GPE without MPII ionic liquid (HNaP-0) was fabricated to compare the results before and after incorporation of MPII ionic liquid. The efficiency (η) was calculated using


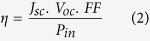


where P_in_ is incident light power, J_sc_ (mA cm^−2^) and V_oc_ (V) are short-circuit current density and open-circuit voltage and FF is fill factor. The fill factor was calculated as


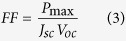


where P_max_ (mW cm^−2^) is the maximum power of solar cell. The DSSC cell was analyzed under Sun simulator with light power of 100 (mW cm^−2^). Figure 5 exhibits the J-V centeracteristic curves of DSSC cells and the inset image shows the prepared free standing GPEs. The GPEs containing MPII ionic liquid show significant enhancement of DSSC efficiency compared with GPE without ionic liquid (HNaP-0). The DSSC parameters are represented in Table 2. The energy conversion efficiencies of 3.94, 4.23, 4.27, 4.80, 5.19 and 5.79% was achieved with HNaP-0, HNaP-1, HNaP-2, HNaP-3, HNaP-4 and HNaP-5 gel polymer electrolytes, respectively. The highest energy conversion efficiency of 5.79% was achieved after incorporation of 100 wt.% of MPII with DSSC parameters namely Jsc (mA cm^−2^), Voc (mV), ff (%) of 13.73, 610 and 69.1 in the HPC:EC:PC:NaI:MPII GPE based DSSCs. The results further indicate that the open-circuit voltage increases after addition of MPII ionic liquid. Consequently, efficiency increases dramatically after the addition of 80 and 100 wt.% of MPII ionic liquid. This can be due to more plasticization effect after the addition of MPII ionic liquid, which results in the highest electron movement and mobility in RNaP-5 followed by increase in the current density of the solar cell. Moreover, the results reveal that open-circuit voltage (Voc) and fill factor (ff) increase with increase in the ionic conductivity which Voc and ff is the highest at HNaP-5 which has the highest ionic conductivity. There is a slight increase in Jsc values with increase in the ionic conductivity. Figure 6 shows effect of MPII ionic liquid on energy conversion efficiency, which maximizes at 100 wt.% of MPII. This work shows significant energy conversion efficiency enhancement of GPE based DSSC with incorporation of MPII ionic liquid.

In conclusion, the gel polymer electrolytes were prepared using EC, PC, NaI and MPII without solvents such as water, acetone, acetonitrile, etc. The highest ionic conductivity of 7.37 × 10^−3^ S/cm was achieved with incorporation of 100 wt.% MPII ionic liquid with respect to the HPC weight. Temperature-dependent ionic conductivity study confirmed that all GPEs follow Arrhenius thermal activated model. Structural and thermal analyses confirmed complexation between HPC, NaI and MPII. XRD results indicated that the intensity of XRD patterns decrease with addition of MPII. Dye-sensitized solar cells were fabricated using HNaP-0, HNaP-1, HNaP-2, HNaP-3, HNaP-4 and HNaP-5 as gel polymer electrolytes and analyzed under Sun simulator in 100 mW/cm^2^. The J-V graph showed energy conversion efficiencies of 3.94, 4.23, 4.27, 4.80, 5.19 and 5.79% using HNa-5, HNaP-1, HNaP-2, HNaP-3, HNaP-4 and HNaP-5 gel polymer electrolytes, respectively. The highest efficiency of 5.79% was achieved with incorporation of 100 wt.% of MPII ionic liquid.

## Methods

### Materials

Hydroxylpropyl cellulose (HPC) (M_w_ ~ 370000), purchased from Aldrich was used without further purification. Sodium iodide salt (NaI) was purchased from Sigma-Aldrich (assay ≥ 99%). The 1-methyl-3-propylimidazolium iodide (MPII) ionic liquid was purchased from Aldrich. The HPC, NaI and MPII were kept dry before use. TiO_2_ P90 (14 nm) and P25 (21 nm) were purchased from AEROXIDE.

### Gel polymer electrolyte (GPE) preparation

The gel polymer electrolytes were prepared through heating and stirring process to gelatinize the HPC. Ethylene carbonate (EC) and propylene carbonate (PC) as plasticizer and iodine (I_2_) as redox mediator were used. The gel polymer electrolytes follow the equation HPC:EC:PC:NaI:xMPII, where x is 20, 40, 60, 80 and 100 wt.% with respect to the HPC weight. Table 1 shows designation of the system. The weights of HPC, EC, PC and NaI were kept at 0.5, 5.0, 5.0 and 0.5 g respectively. The ratio of NaI was optimized (100 wt,% of HPC) before starting the MPII based system. To begin with, the EC and PC was mixed and stirred in a glass bottle and heated at about 100 °C . Subsequently, NaI salt was added into the solution and stirred continuously. MPII ionic liquid was added into the mixture as well. To provide I^−^/I_3_^−^ redox mediator, iodine was added to the mixture where molar ratio of NaI salt and iodine (NaI:I_2_) is 10:1. The mixture was further heated and stirred and HPC was added slowly to the mixture during stirring. The stirring was continued to get a homogenous and gelatinized mixture. After the gel polymer electrolyte was formed, the sample was cooled to room temperature and immediately subjected to electrochemical impedance spectroscopy.

### Dye-sensitized solar cell (DSSC) fabrication

Photo-electrode layer was prepared with two TiO_2_ layers on the FTO substrate. The first layer was prepared using the spin coating technique. At the first layer which was spin coated, 0.5 g of TiO_2_ (P90) was grounded for 30 minutes with 2 ml HNO_3_ (PH = 1) in agate mortar and spin coated on FTO at 1000 rpm for 2 seconds followed by 2350 rpm for 60 seconds. Finally, the substrate was sintered at 450 °C for 30 min. For the second layer, 0.5 g of TiO_2_ (P25) was grounded for 30 minutes with 2 ml HNO_3_ (PH = 1) in an agate mortar with 1 drop of Triton X-100 and 0.1 g carbowax. The solution was then doctor bladed on the first layer and sintered at 450 °C for 30 min. After the substrate cooled down to room temperature, the photo-electrode was soaked in N719 dye solution for 24 hrs. Counter electrode was prepared using commercial platinum solution and coated on FTO substrate and sintered at 450 °C for 30 min. The prepared GPEs were sandwiched between two electrodes and analyzed under Sun simulator.

### Characterization

For ionic conductivity and temperature-dependent conductivity study, the GPEs were studied using electrochemical impedance spectroscopy (EIS), Hioki, 3532-50 LCR HiTESTER. The XRD patterns of GPEs were recorded using PANalytical Empyrean diffractometer (45 kV, 40 mA) with Cu-Kα radiation and wavelength of λ = 1.540600 Ǻ for 2*θ* range of 5–80° at ambient temperature. The GPEs were subjected to thermal studies using simultaneous thermal analyzer (STA) PERKIN ELMER (STA 6000). The Nitrogen flow rate was 20 ml/min. The GPEs were heated with ramping rate of 10 °C/min from 25 to 600 °C to obtain decomposition temperature (T_dc_). Gel polymer electrolytes were further analyzed using differential scanning calorimetry (DSC) using NETZSCH DSC 200 F3 with liquid nitrogen cooling system. The experiment was performed under a nitrogen flow rate of 50 ml/min. The GPEs were sealed in the aluminum pan and analyzed in heat-cool-heat process within 4 cycles with heating and cooling rate of 10 K/min. In the first cycle, the sample was cooled from room temperature to −140 °C. In cycle 2, the heating process was performed from −140 °C to 130 °C. In cycle 3, the cooling process was applied from 130 °C to −140 °C. In Cycle 4, the final cycle, heating process was applied from −140 °C to 130 °C. At the end of each cycle, an isothermal process was applied for 1 minute. Cycle 2 was used to measure glass transition temperature (T_g_).

## Additional Information

**How to cite this article**: Khanmirzaei, M. H. *et al.* Hydroxypropyl Cellulose Based Non-Volatile Gel Polymer Electrolytes for Dye-Sensitized Solar Cell Applications using 1-methyl-3-propylimidazolium iodide ionic liquid. *Sci. Rep.* **5**, 18056; doi: 10.1038/srep18056 (2015).

## Figures and Tables

**Table 1:** Designation and ionic conductivity of GPE system.

Designation	HMII ionic liquid (wt.%)	Conductivity, σ (S cm^−1^)
HNaP-0	0	4.94 × 10^−3^
HNaP-1	20	5.85 × 10^−3^
HNaP-2	40	5.79 × 10^−3^
HNaP-3	60	5.93 × 10^−3^
HNaP-4	80	6.49 × 10^−3^
HNaP-5	100	7.37 × 10^−3^

**Table 2:** Dye-sensitized solar cell parameters for HPC:EC:PC:NaI:MPII GPE system.

Electrolyte	J_sc_ (mA cm^−2^)	V_oc_ (mV)	FF (%)	Efficiency, η (%)
HNaP-0	13.65	495	58.3	3.94
HNaP-1	11.69	580	62.4	4.23
HNaP-2	12.72	550	61.0	4.27
HNaP-3	13.20	575	63.3	4.80
HNaP-4	14.71	565	62.5	5.19
HNaP-5	13.73	610	69.1	5.79
